# Physical activity profile of Nigeria: implications for research, surveillance and policy

**DOI:** 10.11604/pamj.2018.30.175.12679

**Published:** 2018-06-26

**Authors:** Adewale Luqman Oyeyemi, Adetoyeje Yoonus Oyeyemi, Babatunji Abayomi Omotara, Aliyu Lawan, Kingsley Kolapo Akinroye, Rufus Adesoji Adedoyin, Andrea Ramírez

**Affiliations:** 1 Department of Physiotherapy, College of Medical Sciences, University of Maiduguri, Nigeria; 2 Department of Community Medicine, College of Medical Sciences, University of Maiduguri, Nigeria; 3 Nigerian Heart Foundation, Lagos, Nigeria; 4 Department of Medical Rehabilitation, College of Health Sciences, Obafemi Awolowo University, Nigeria; 5 Post-Graduate Program in Epidemiology, Federal University of Pelotas, Pelotas, Brazil

**Keywords:** Physical inactivity, non-communicable diseases, health promotion, public health

## Abstract

Appraising the status of physical activity surveillance, research and policy in Nigeria is relevant to national and regional public health actions on physical activity promotion and non-communicable disease control. This study aimed to 1) evaluate the physical activity profile of Nigeria and 2) propose strategies for improving physical activity in the country. The Global Observatory for Physical Activity-GoPA! with inputs from local experts systematically collected sociodemographic and physical activity surveillance, national policy and research indicators data for Nigeria in 2014. The Nigerian Country Card highlighting the status of these indicators was developed in 2015 and launched in 2016. Prevalence of physical activity among Nigerian adults was 78% (female=76%, male=79%). There was no physical activity surveillance system and national plan, and no empirical data on the proportion of all deaths directly due to physical inactivity in Nigeria. Few (n=7) articles related to physical activity and public health were published in 2013 and the country occupied the 38^th^ position in the global research ranking, contributing about 0.24% to physical activity research worldwide. Implementing national physical activity plans and multi-sectorial collaborations between government and non-governmental partners are needed to improve physical activity surveillance, research and policy in Nigeria and other African countries with similar physical activity gaps.

## Introduction

Physical inactivity-related non-communicable diseases (NCDs) are responsible for about 3 million deaths in sub-Saharan Africa [1]. These deaths are expected to increase up to 80% by 2020 if urgent actions are not taken [1-3]. In Nigeria, NCDs already account for at least one quarter and one third of all deaths in males and females, respectively [4]. Improving physical activity level is a recommended strategy for controlling NCDs [1]. However, for effective country specific intervention, it is important to first establish the status of surveillance system, national policy and research capacity on physical activity. Until now, there was no observatory dedicated to monitor physical activity at the country level and worldwide. The Global Observatory for Physical Activity-GoPA! was established to develop a physical activity card that could facilitate the need for country level data collection and monitoring to inform policy and planning of interventions at the population level for each country of the world [5,6]. Nigeria is the sixth largest country of the world and the most populated in Africa [7]. Thus, tracking the physical activity profile of this country is relevant to national, regional and international public health actions. Specifically, evaluating the physical activity profile of Nigeria could help identify research, surveillance and policy gaps and provide information on data needed for effective public health planning and action in the country. The aims of this study were to 1) evaluate the physical activity profile of Nigeria and 2) propose strategies for improving physical activity in the country.

## Methods

The Nigerian Country Card (physical activity profile) was systematically created as part of the first set of physical activity country cards developed by the Global Observatory for Physical Activity-GoPA! in 2014. The methods and design of the GOPA! study have been described fully elsewhere [5,6,8]. Briefly, using a standardized methodology and indicators (to warrant comparability over time), GoPA! in 2014 collected data on country characteristics, surveillance, national policy, and research metrics for the world countries and summarized them in accessible and all-inclusive public physical activity country profiles called the “Country Cards”. Physical activity data and statistics were collected from recognized and acknowledged sources of information including the World Health's organization Global Health Observatory Data [9], World Bank [10-13], United Nations [14]. The Lancet 2012 physical activity series [15] and PubMed. Country contacts were selected based on a PubMed search of the physical activity literature, supplemented by recommendations from public health experts. All country contacts demonstrated experience in the area of physical activity and public health either as researchers or as members of government institutions. Nigeria's Country Card was reviewed and revised by the country's representative and a working group (including researchers and non-academics with experience in physical activity, public health and advocacy), in order to ensure that the most recent and accurate information available up to 2013 was presented. Through this process, the Nigerian physical activity profile was created to highlight the status of national progress in promoting physical activity among adults (age 18+ years), and was launched in 2016 as part of the 140 country cards in the first GoPA! Physical Activity Almanac. The data sources and profile descriptors for each of the five indicators (demographic, health burden due to physical inactivity, physical activity prevalence, surveillance and policy status and research characteristics) of the Nigerian country card are summarized in Table 1. The detailed description of scoring methods and calculations of the estimated values for the indicators are available on the GOPA's website [16].

**Table 1:** Summary of indicators and data sources for the Nigerian Country profile

Indicators	Profile descriptors	Data sources
Demographic	Total population	World Bank, country data
	Life expectancy	World Bank, country data
	GINI inequality index	World Bank, country data; CIA’s World Factbook
	Literacy rate	World Bank, country data; CIA’s World Factbook; WHO Global Health Observatory data
	Human development index	International Human Development Indicators, United Nations
Mortality	Deaths by non-communicable diseases	World Bank
	Deaths related to physical inactivity	Lee et al.^15^ The Lancet 2012 Physical Activity Series
Physical activity prevalence	Prevalence	WHO Global Health Observatory data
Surveillance and policy	Physical activity plan	WHO MiNDbank database of resources; Google and PubMed; country contact
	National survey	Demographic & Health Survey website; WHO database; Google search; country contact
Research characteristics	Articles related to physical activity and public health	PubMed
	Number of active researchers	PubMed
	Average connection among authors	PubMed
	Identified publishing groups	PubMed
	Researchers per million people	PubMed and World Bank
	Articles per million people	PubMed and World Bank
	Ranking (country contribution to physical activity research worldwide in 2013)	PubMed and World Bank

## Results

**Figure 1:**
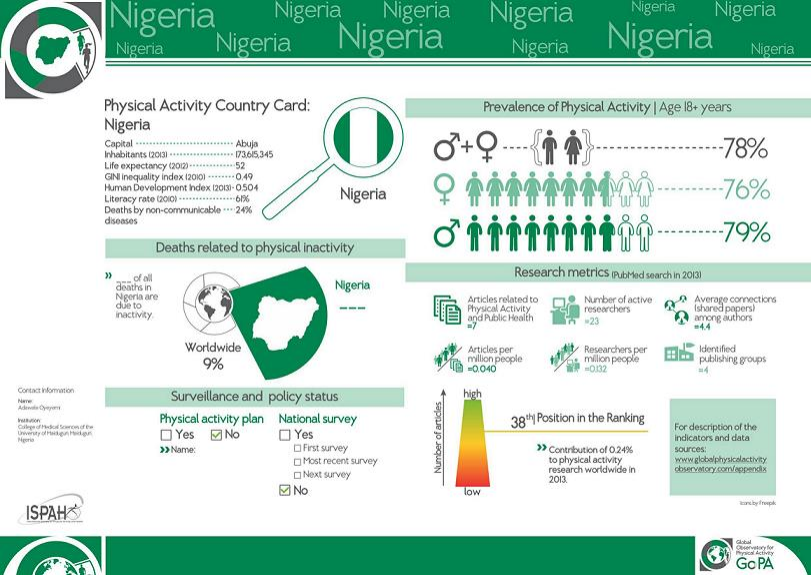
The Nigerian country card of physical activity profile (the global observatory for physical activity, 2016)

The results for the Nigerian Country profile on the five indicators about physical activity are shown in Figure 1.

**Demographic indicator**: Nigeria is an African lower middle income country with approximately 182 million inhabitants. The country's GINI inequality index and human development index were 0.49 and 0.504, respectively. NCDs are responsible for 24% of all deaths in Nigeria.

**Mortality due to physical inactivity indicator**: No data exists on the proportion of all deaths directly due to physical inactivity in Nigeria.

**Physical activity prevalence indicator**: The prevalence of physical activity among Nigerian adults was 78% (95% CI=33.7-91.5). Prevalence was higher among males=79% [95% CI=32.6-90.7]) than females=76% [95% CI=33.3-91.6].

**Surveillance and policy status indicator**: There was no physical activity surveillance system in place to monitor physical inactivity in Nigeria. Also, no national physical activity plan exists for Nigeria.

**Research characteristics indicator**: Seven articles related to physical activity and public health were published for Nigeria in 2013. The country occupied the 38^th^ position in the global research ranking, contributing about 0.24% to physical activity research worldwide. Only 23 active researchers on physical activity and about 4 publishing research group were available in Nigeria as of 2013.

## Discussion

There is no data on the prevalence of deaths due directly to physical inactivity in Nigeria. However, a population prevalence of about one-quarter of all deaths due to physical inactivity-related NCDs is an urgent call to prioritize physical activity as a public health agenda in Nigeria. To scale up effective physical activity interventions against NCDs in Nigeria, there is a need to develop bold initiatives and implement policies that will increase physical activity across all sectors including transportation, urban planning, sports and recreation and workplaces and schools. Although the proportion of Nigerian adults (78%) that met the health recommendations of at least 150 minutes of moderate-intensity or 75 minutes of vigorous-intensity physical activity per week, or an equivalent combination [17] was more than the global average of 76.7% [18], about 22% of adults' population in Nigeria still remain inactive. Moreover, it should be noted that the present physical activity estimate for Nigeria was from a subnational study conducted about 2 decades ago [19]. Thus, the Ministry of Health in Nigeria should prioritize the implementation of a national physical activity survey for accurate prevalence estimate for the country. Also, our finding reflects the need to institute a surveillance system for monitoring physical activity at the population level in Nigeria. Due to the public health relevance of physical activity monitoring to NCDs, the Federal Ministry of Health should partner with actors at the National Population Commission to integrate physical activity as part of the key national health indicators routinely collected and reported in the Nigeria Demographic Health Survey (DHS) [20].

The DHS represents a first gap cost effective and feasible approach for surveillance and monitoring of population level physical activity in Nigeria. An alternative that could be more enduring for effective physical activity surveillance is for the Ministry of Health to incorporate the WHO STEPwise approach to Surveillance (STEPS) survey on NCD risk factors into the national health reporting system of Nigeria. The Lack of national plan or policy on physical activity in Nigeria could undermine government capacity to mitigate the epidemic of physical inactivity-related NCDs in the country. Although a NCDs policy draft recently exits for Nigeria [21], its implementation has not been evaluated and intervention plans for physical activity were not pragmatically embedded in the policy document. National public health action to reduce population physical inactivity is best served when physical activity promoting policies are stand-alone or strategically defined in national public health plans [5]. However, physical activity plans for Nigeria should include resources and mechanisms on how to best engage multiple partners and stakeholders (e.g, health and social care professionals, urban and transportation planners, sport and recreation providers, educators, policy makers and the non-governmental organizations) on policy formulation and implementation. For example, policies to improve population level of active transportation-physical activity in Nigeria, should not only focus on population mobilization through health education but also target multilevel factors including provision of safe and adequate infrastructures to support walking and bicycling and implementing traffic control measures that reduce pedestrian exposure to high traffic volume and speed.

Few investigators were actively engaged in physical activity research in Nigeria in 2013, indicating the need to build capacity and research workforce in the country. A viable strategy that can be used to improve physical activity research in developing countries is to create more graduate-level programme to train researchers in physical activity and public health [22]. For Nigeria and other developing African countries, it is timely to introduce courses on physical activity in the curriculum and training of medical and health professional students. This could facilitate the creation of the needed workforce of professionals to advance physical activity research in the region countries. Further, it is important that leading physical activity researchers in Nigeria collaborate to establish physical activity society or pressure group with cross-sectorial membership, including health and non-health professionals. Improving physical activity research and population level participation in Nigeria is ultimately a local responsibility that could be better served through the commitments of dedicated actors from various sectors including the government. The limitation of our study includes the non-representativeness of the physical activity prevalence estimate for Nigeria; it was derived from a subnational sample of adults in only one of the 37 states of the country [19]. Also, the review of evidence for the research characteristic indicator was done using mainly PubMed search. It is possible that many evidence relevant to Nigeria was omitted using this approach. However, other available country cards for 140 countries used similar search strategy making the process comparable worldwide.

## Conclusion

This study highlights the need to improve the physical activity status of Nigeria. The Nigerian physical activity card represents a unique tool that can be used to sensitize government, non-governmental organizations and researchers on the dearth of information on physical activity monitoring, research and intervention and the need to bridge the policy gaps on physical activity plans in Nigeria. Implementing physical activity policy or national plan, establishing physical activity workforce in public health, integrating physical activity surveillance into the Nigeria Demographic Health Survey and supporting multi-sectorial collaborations are feasible and best buy strategies that can help Nigeria move towards a more physically active population and society. These strategies can also be adopted by other African countries with similar gaps in physical activity profile.

### What is known about this topic

- Physical inactivity is a global public health problem;
- Physical inactivity-related NCDs are increasing in the low-and middle-income countries including Nigeria;
- National status and strategies for promoting physical activity are unreported for African countries including Nigeria.

### What this study adds

- There is large gap in surveillance, research, policy and national plan on physical activity in Nigeria;
- There is urgent and compelling needs to make the population and society more physically active in Nigeria;
- Creating physical activity workforce in public health, supporting multi-sectorial collaborations, integrating physical activity surveillance in existing national demographic and health reporting system and implementing pragmatic national policies and plans on physical activity are feasible strategies to adopt to improve the physical activity status of Nigeria.

## Competing interests

The authors declare no competing interests.
